# Chemokine Heterocomplexes and Cancer: A Novel Chapter to Be Written in Tumor Immunity

**DOI:** 10.3389/fimmu.2018.02185

**Published:** 2018-09-25

**Authors:** Gianluca D'Agostino, Valentina Cecchinato, Mariagrazia Uguccioni

**Affiliations:** ^1^Laboratory of Chemokines in Immunity, Institute for Research in Biomedicine, Faculty of Biomedical Sciences, Università della Svizzera italiana, Bellinzona, Switzerland; ^2^Department of Biomedical Sciences, Humanitas University, Milan, Italy

**Keywords:** chemokines, tumor microenvironment, heterocomplexes, CXCL12, HMGB1

## Abstract

Infiltrating immune cells are a key component of the tumor microenvironment and play central roles in dictating tumor fate, either promoting anti-tumor immune responses, or sustaining tumor growth, angiogenesis and metastasis. A distinctive microenvironment is often associated to different tumor types, with substantial differences in prognosis. The production of a variety of chemotactic factors by cancer and stromal cells orchestrates cell recruitment, local immune responses or cancer progression. In the last decades, different studies have highlighted how chemotactic cues, and in particular chemokines, can act as natural antagonists or induce synergistic effects on selective receptors by forming heterocomplexes, thus shaping migratory responses of immune cells. A variety of chemokines has been described to be able to form heterocomplexes both *in vitro* and *in vivo* under inflammatory conditions, but nowadays little is known on the presence and relevance of heterocomplexes in the tumor microenvironment. In recent years, the alarmin HMGB1, which can be massively released within the tumor microenvironment, has also been described to form a complex with the chemokine CXCL12 enhancing CXCR4-mediated signaling, thus providing an additional regulation of the activity of the chemokine system. In the present review, we will discuss the current knowledge on the synergy occurring between chemokines or inflammatory molecules, and describe the multiple functions exerted by the chemokines expressed in the tumor microenvironment, pointing our attention to the synergism as a possible modulator of tumor suppression or progression.

## Introduction

The leukocyte infiltrate is a key component of the cancer stromal compartment. Within the tumor, the wide range of chemokines produced by both malignant and stromal cells can affect the composition and the phenotype of the cell infiltrate, and influence tumor growth, survival and metastasis (1–5). Chemokines that regulate leukocyte migration and play key roles in both physiology and pathological conditions (6–8), are small proteins of 8–12 KDa, which can be divided into 4 groups (CCL-, CXCL-, CX3CL1, and XCLs chemokines) according to the position of two conserved cysteine residues within their structure. The chemokine system is characterized by a set of almost 50 ligands, which engage in a promiscuous fashion a panel of more than 20 chemokine receptors, including conventional and atypical receptors, expressed by immune cells, endothelial cells and cancer cells (9–12). The promiscuous pattern of interaction, together with the large number of ligands and receptors, enables the chemokine system to mediate a variety of cell functions. This is of particular relevance in tumors, since chemokines can influence angiogenesis, cell-adhesion, cell extravasation, and survival (7). Different chemokines can also interact together showing antagonistic or synergistic activity on specific chemokine receptors. They can trigger simultaneously different receptors, resulting either in the inhibition or in the enhancement of the intracellular cell signaling (13, 14), or a single receptor can be activated by a heterocomplex formed between two chemokines, resulting in a stronger cellular response (13, 15). Additionally, chemokines can also interact with inflammatory mediators released in the microenvironment, amplifying cellular responses induced by chemokine receptors (16, 17).

While the multiple roles of heterocomplexes in the early stage of inflammation and in regeneration have been clearly dissected (13, 18, 19), little is known about their functions in tumors (20), and further studies are necessary to define their significance. Von Hundelshausen and colleagues have performed a thorough study mapping the chemokine heterocomplexes by bidirectional immunoligand blotting (21). This study opens the debate on the *in vivo* relevance of the multitude of heterocomplexes found *in vitro*. In the present review, we discuss examples on how the concomitant expression of several chemokines with either anti- or pro-tumor functions could favor heterocomplexes formation in the tumor microenvironment (TME), thus adding an additional feature to be considered in tumor immunity.

## Chemokine heterocomplexes

Several studies in the last decade have described the heterodimerization between chemokines as a regulatory mechanism that governs their activity under inflammatory conditions. In the TME, chemokines play crucial roles either favoring immune responses against the tumor or promoting cancer progression and metastasis. Of note, similar chemokine expression profiles can result in a different tumor-specific leukocyte infiltrate. This phenomenon suggests that additional regulatory mechanisms might be involved, including the release of proteins able to modify chemokine activity. It is now well established that a chemokine receptor can be triggered by a low concentration of its selective agonist when a synergy-inducing chemokine, not selective for the receptor but able to form a complex with the agonist, is concomitantly present (17, 22, 23) (Table 1). The first evidence of the synergism induced by the presence of two chemokines was provided by Struyf and colleagues (24), who described the synergy between Regakine-1 and CXCL8, and between Regakine-1 and CCL7. Few years later, the same group has shown that CXCL8 can enhance CXCL12 responses and this enhancement is CXCR4 mediated (25). In 2005, three groups described the formation of heterocomplexes between chemokines, able to enhance the activity of CCR7, CCR4, CCR5, and CXCR2 (23, 26, 29, 30). In particular, CXCL13 forms a complex with CCL19 and CCL21, leading to CCR7 activation at lower agonist concentrations (23). The CXCR3- and CCR4-agonists, CXCL10 and CCL22, co-expressed in the inflamed skin, synergistically interact together, through the first β-strand of CCL22, enhancing CCR4-mediated chemotaxis of T cells, independently from CXCR3 or GAGs binding (29). Other studies showed that the heterocomplex between CCL5 and CXCL4, formed through heterophilic interactions, plays a crucial role in triggering monocyte arrest on the endothelium (30). In this case, the authors demonstrated the requirement of GAGs on the cell surface, and that the CCL5/CXCL4 complex showed paired N-terminus, resembling a CC-type heteromer that promoted a more efficient receptor activation (30, 31). NMR spectroscopy and molecular modeling, followed by *in vitro* analysis, have shown the structure of a heterocomplex between CXCL8 and CXCL4. This complex was shown to enhance the anti-proliferative effect of PF4 on endothelial cells, and the CXCL8-induced migration of CXCR2 transfected cells (26).

**Table 1:** Synergy-inducing chemokines heterocomplexes.

**Receptor**	**Heterocomplexes**	**Synergistic Functions**	**References**
CXCR1/2	CXCL8/Regakine-1 CXCL8/CXCL4 CXCL7/Regakine-1	Chemotaxis of neutrophils and of CXCR1-transfected Jurkat cells.	(24–27)
CCR7^*^ CXCR5	CCL19/CXCL13 CCL21/CXCL13 CXCL13/CCL19 CXCL13/CCL21	Chemotaxis of CCR7+ transfected PreB cells and human leukocytes (DCs, B and T lymphocytes). Increased chemotaxis of CCR7+ Sezary Syndrome (SS) cells.	(23, 28)
CCR4^**^	CCL22/CXCL10 CCL22/CCL19 CCL17/CXCL10 and many others	Chemotaxis of human T lymphocytes (Th1-Th2).	(29)
CCR5	CCL5/CXCL4	Triggering of monocytes arrest on activated endothelium under flow conditions. Blockade of CCL5/CXCL4 heterocomplex inhibits atherosclerosis in hyperlipidemic mice.	(30, 31)
CCR2	CCL2/CCL19 CCL2/CCL21 CCL7/CCL19 CCL7/CCL21 CCL7/Regakine-1	Induction of chemotaxis and responses in monocytes and lymphocytes.	(24, 32)
CXCR4	CXCL12/CXCL9	Recruitment of tumor-infiltrating lymphocytes in primary central nervous system lymphoma.	(20)
CXCR4	CXCL12/HMGB1	Promotion of monocytes chemotaxis both *in vitro* and *in vivo*. Tissue regeneration (liver, muscle, bone).	(16, 18, 19)

* Additional CXC and CC chemokines have been shown in this study to act in synergy with the selective CCR7 agonists. Migration is enhanced in human mature dendritic cells, B cells, T cells, and CCR7-transfected cells.

** *Additional CXC and CC chemokines have been shown in this study to act in synergy with CCL22. Migration is enhanced in CCR4-transfected cells*.

Later on, the CCR7-agonists, CCL19 and CCL21, were described as enhancer of monocytes recruitment by forming heterocomplexes with CCL7 and CCL2, resulting in an augmented CCR2 response, and preventing CCL7 and CCL2 degradation by ACKR2 (32).

A study directly supporting the idea that the activity of heterocomplexes can be relevant also in cancer was performed in our laboratory, showing the role of the CXCL9/CXCL12 heterocomplex in primary central nervous system lymphoma (PCNSL). In this work, it was shown that CXCL9 and CXCL12 are co-expressed in the perivascular area of the tumor, and can form a complex enhancing CXCR4-mediated recruitment of tumor-infiltrating lymphocytes and malignant B cells. This synergism might serve as regulator of the recruitment of CD8+/CXCR4+/CXCR3+ T cells and CXCR4+/CXCR3- malignant B cells in the perivascular cuffs, forming the typical lesions of these tumors (20).

## CXCL12/HMGB1 heterocomplex

A synergism, mediated by the heterocomplex formed between CXCL12 and the DAMP protein HMGB1 has been shown to be relevant in monocyte recruitment (16, 33) and in tissue regeneration (19, 34). However, its involvement in modulating tumor progression and metastasis has never been assessed. Nonetheless, both CXCL12 and HMGB1 are key players in the TME, where they orchestrate a variety of functions that sustain cancer progression. Indeed, the CXCL12/CXCR4 axis is hyper-activated in lymphomas and in many solid tumors. Their activity is central in the promotion of tumor progression and metastasis to the lungs, brain and bone (35, 36). HMGB1 plays a variety of functions based on its cellular location: in the nucleus, is essential for nucleosomes dynamics and chromosomal stability; in the cytosol or mitochondria, modulates autophagy and apoptosis and regulates mitochondrial morphology and functions; on the cell surface of neurons, promotes axon sprouting and neurite outgrowth (37). Stressed and cancer cells release HMGB1 in the extracellular space, where it activates different receptors in a redox-sensitive manner. The disulphide-HMGB1 promotes TLR-4 activation and mediates production of inflammatory cytokines and chemokines, whereas the reduced-HMGB1 triggers RAGE to promote autophagy and CXCL12 secretion. The reduced form is also able to complex with CXCL12 mediating CXCR4-dependent chemotaxis (33, 38). The CXCL12/HMGB1 heterocomplex acts as an enhancer of CXCR4-mediated signaling, potentiating ERK activation, calcium rise and chemotaxis, both *in vitro* and *in vivo* (17). The effect can be blocked by glycyrrhizin and by anti-CXCL12 antibodies, which prevent the formation of the heterocomplex, or by AMD3100, proving the sole involvement of CXCR4 (16, 39). Moreover, the heterocomplex induces a rearrangement of the N-terminus of CXCL12 and conformational changes in the CXCR4-dimers (16) that might suggest a different mode of receptor triggering.

Recently an important role of the CXCL12/HMGB1 heterocomplex has been described in tissue regeneration. Fully reduced HMGB1 promotes liver and muscle regeneration through CXCR4, by acting on muscle stem cells, hepatocytes, and infiltrating cells (18). In a similar study, HMGB1 was detected after fracture both in humans and in animal models, and the heterocomplex acting via CXCR4 promotes *in vivo* skeletal, hematopoietic and muscle regeneration (19).

## Chemokine functions in the tumor microenvironment

During the different phases of cancer progression, many types of inflammatory cells that exhibit either anti- or pro-tumoral functions are recruited from the blood stream by specific chemotactic cues. The leukocyte infiltrate includes neutrophils with different phenotypes (40), macrophages (41), natural killer cells (NK) (42), myeloid-derived suppressor cells (MDSC) (43, 44), dendritic cells (DCs) (45), T and B lymphocytes (46, 47). Several chemokines have been shown to be expressed in tumors, guiding leukocyte recruitment and positioning, and to support tumor spread at distal organs (7). Below we provide some examples in which different cell types present in tumors can be recruited in the TME thanks to the activity of chemokines, and possibly to the presence of heterocomplexes.

### Anti-tumoral functions

Chemokines mediate anti-tumor activities through the recruitment of specific immune cell types (48). CXCL9 and CXCL10, agonists of the CXCR3, promote the recruitment of CD4+ Th1 lymphocytes, NK cells, and CD8+ cytotoxic T lymphocytes (CTL) to the TME, where they exert a potent anti-tumor activity (7, 49). Th17 cells further sustain the recruitment of CTL, NK cells (50), and DCs (51). In particular, CTL specific for tumor-associated antigens (TAA), together with Th1 and NK cells expressing IFNγ, guide immunity against the tumor promoting tumor cell apoptosis, and releasing effector cytokines and cytotoxic molecules (48). Indeed, evidence in patients with ovarian cancer demonstrated that the increased expression of CXCL9 and CXCL10 correlates with an increased number of tumor-infiltrating CTL and a high CD8+/regulatory T cells ratio that lead to a reduction in cancer metastasis and to a better prognosis (52). IFNγ produced within the TME induces CXCL9, CXCL10, and CXCL11 expression, which correlates with tumor infiltrating CTL and Th1-effector cells and with a positive survival rate in colorectal cancer (53). Moreover, the presence of CTL, CXCL9, and CXCL10 within the tumors is associated to a positive response to PD1/PD1L blocking therapies (54, 55). In recent years, Bronger and colleagues demonstrated that CXCL9 and CXCL10 expression can predict survival in high-grade serous ovarian cancer patients (56).

Tumor tissues from ovarian cancer patients show a dynamic T cell infiltration at different disease stages. Th17 and Th1 cells are present in the early stages, associated with an anti-tumor immune response and production of CXCL9 and CXCL10 (56). In the later stages Treg, expressing CCR4, correlate with CCL22 production, and are associated to pro-tumoral immunosuppressive functions (57). The role of the CCL22/CXCL10 heterocomplex (29) in the switch from an anti- to a pro-tumoral TME should be investigated.

CXCL9 and CXCL12 can form heterocomplexes, and in PCNSL are coexpressed on the tumor vasculature. CXCL12-induced migration is enhanced in CXCR4+/CXCR3+/CD8+ T lymphocytes and in CXCR4+/CXCR3− malignant B cells, indicating that chemotactic cues in the perivascular environment serve as regulators for the recruitment of tumor infiltrating lymphocytes (TILs) (20). Tumor associated macrophages (TAM) are also a source of CXCL9 and CXCL12. Interestingly, the expression of CXCL9 is restricted to macrophages present in the perivascular area, indicating heterogeneity among macrophages within the tumor, and suggesting this cell type as the most important player for the recruitment of CTL in the perivascular space (20).

TAM, recruited to the tumor in response to chemokines, polarize toward different subtypes (M1 or M2) accordingly to the presence of activating stimuli generated by the cytokines expressed in the microenvironment. M1 macrophages produce CXCL9 and CXCL10 and exert an anti-tumoral activity, while M2 macrophages sustain cancer growth (5, 41, 58). CXCR3 agonists are also important for the polarization toward a M1 phenotype, since CXCR3 deficiency of this receptor induces a M2 phenotype (59).

Tumor associated neutrophils (TAN) polarized toward a N1 phenotype exert an anti-tumoral activity. In particular, TGF-β blockade increased neutrophil attracting chemokines (CXCL2, CXCL5, CCL3) specific for CXCR1/2 and CCR2-5. This resulted in an influx of CD11b+/Ly6G+ TAN with enhanced tumor cytotoxic activities and higher levels of pro-inflammatory cytokines (60).

The expression of CCR5 on CD4+ and CD8+ T lymphocytes has been described to be essential for an efficient tumor rejection in mouse model of Lewis lung adenocarcinoma and pancreatic adenocarcinoma (61). The activity of CCL5, a selective CCR5 agonist, can be enhanced by CXCL4 (30), a chemokine expressed by a variety of tumor types (62). Interestingly, in both tumor types the expression of CXCL4 have been documented (62), and could represent an additional tool for enhancing CCR5 responses.

The recruitment of other cell types including DCs and B cells with antigen presenting functions is essential for the expansion and activation of leukocytes within the TME (48). High levels of B cell-infiltrates, recruited into the microenvironment through the CXCL12/CXCR4 axis, are positively associated with a good survival rate in breast cancer, high-grade serous ovarian cancer, and cervical cancer (63–65). B cells infiltrating the tumor can organize in tumor-associated tertiary lymphoid structures, where they act as antigen presenting cells enhancing T cell responses or producing tumor-specific antibodies (66). In breast cancer, a specific subset of T follicular helper cells, through the production of CXCL13, has been linked to tertiary lymphoid structures formation, generation of germinal centers and maturation of B cells, migrating into the TME via CXCR5 (67).

A recent study showed that NK cells, through the production of CCL5 and XCL1, recruit DCs into the TME promoting cancer immune control, which is associated with patient survival (68).

### Pro-tumoral functions

Chemokines can also support tumor progression and metastasis, either acting as angiogenic factors (69), or through the recruitment of different immune cell types into the TME, which inhibit effector cell functions (7, 48).

Within the CXC-family of chemokines, an important role in inducing angiogenesis has been demonstrated for chemokines containing the ELR motif (glutamic acid-leucine-arginine). Neovascolarization is an essential process that sustains solid tumor growth and metastasis. In humans, CXCR2 is considered the receptor mainly involved in angiogenesis through the interaction with ELR+ chemokines (CXCL1, CXCL2, CXCL3, CXCL5, CXCL6, CXCL7, and CXCL8). CXCR2 activity has been directly correlated with the aggressiveness of a number of tumors, including melanoma (70), pancreatic cancer (71), gastrointestinal cancers (72), and renal cell carcinoma (73).

CCL22 and CCL28, expressed in many human tumors, are mediators for the recruitment of CCR4+/CCR10+ Treg cells, involved in the suppression of both spontaneous and therapy-induced local tumor immunity. The presence of these cells is associated to a poor prognosis (74–76). It has been demonstrated also that Treg directly support angiogenesis through the secretion of VEGF and promote metastasis via the induction of NK cells apoptosis (75, 77). Interestingly, the expression of CXCR3 by Treg resulted in an immunosuppressive effect mediated by the control of Th1-associated responses (78). In addition, Treg with a memory phenotype are frequently recruited through CXCR4/CXCL12 signaling to the bone marrow, a common target of metastasis in humans, further supporting the idea that this cell subset provides an anti-inflammatory environment that sustains cancer progression (79, 80).

Th22 cells, that under physiological conditions express CCR10, CCR6 and CCR4, and home to the skin (81), have been shown to be recruited to the tumor site, supporting tumorigenesis through the activation of STAT3 and the enhancement of the expression of the methyltransferases DOT1L (82) and of the Polycomb repressive complex 2 (PRC2) (83). B cells, as well, can exert a regulatory function by inhibiting T cells activity through the production of TGF-β and IL-10, or further support tumorigenesis via the production of TNF (84, 85). Their recruitment to the tumor sites is mediated by the CXCL12/CXCR4 axis, and might be enhanced by the chemokines known to form a complex with CXCL12.

Myeloid-derived suppressor cells (MDSCs) are deeply investigated in tumor models and in cancer patients, due to their relevant role in promoting cancer stemness (43, 44). Granulocytic MDSCs, mainly composed by different subsets of neutrophils, express CXCR1 and CXCR2, and are recruited to the tumor by CXCL8, produced by tumor cells or by Treg (86). In the TME, they release molecules that sustain angiogenesis, further supporting tumor progression and metastasis (44). Interestingly, CXCL8 has been shown to synergize with CXCL4, which is produced by a variety of tumors at different stages (62). Monocytic MDSCs, that include macrophages at different maturation stages, express CCR2, CXCR2 and CXCR4, and can reach the tumor via their specific ligands CCL2, CXCL5 and CXCL12 respectively (87, 88). These cells are able to sustain tumor growth via the induction of arginase-I, iNOS, and TGF-β, and favor the recruitment of Treg at the tumor site through the production of CCR5-binding chemokines (89).

The M2 subset of TAM is negatively correlated with survival in cancer, and is associated with responses that sustain tumor growth and progression (41, 90).

Plasmacytoid DCs can reach the TME via the CXCR4/CXCL12 axis. Their recruitment sustains tumor growth by the induction of IL-10 producing Treg that in turn suppress the activation of tumor specific effector T cells (91, 92). As shown by Vanbervliet and colleagues, the sensitivity of this cell type to CXCL12 can be enhanced by the CXCR3 agonists (93). Nonetheless, this type of synergy was interpreted as the activity of both CXCR4 and CXCR3, and was not demonstrated if this effect was due to a heterocomplex formation, as shown later in the PCNSL (20).

## Conclusions

Many chemokines are abundantly and concomitantly expressed in the TME and orchestrate a variety of functions that sustain cancer progression or suppression. While the activity of chemokine heterocomplexes has been deeply investigated in inflammatory conditions, and in models of tissue regeneration, a direct prove that a heterocomplex can enhance the responses of tumor cells to chemokines has been demonstrated only for the CXCL12/CXCL9 heterocomplex in PCNSL (20). The concepts covered in the present review suggest that the nature and function of tumor infiltrating immune cells might not be the simple result of the interaction occurring between a chemokine agonist and its specific receptor, but, could be mediated by chemokine heterocomplexes that can differently modulate the activation of a variety of chemokine receptors regulating cell recruitment, positioning, and the switch in the components of the cellular infiltrate in different tumor stages.

The mapping of the possible chemokine-chemokine interactions by bidirectional immunoligand blotting suggests that the synergism might preferentially be mediated by CC-type heterodimers, whereas the CXC-types might promote inhibitory effects (21). Additional studies are required to determine whether this distinction can be applied to the whole chemokine system, and in particular if the heterocomplexes identified are relevant in the TME.

As testified by the diverse expression of chemokine receptors in tumors and by the multiple activities of the heterocomplexes studied so far, we might expect different responses to the same heterocomplex according to the distinctive features of each TME. A deeper understanding of the modulation of the chemokine system in TME, will tell us the relevance of the heterocomplexes, and their possible involvement in shaping the activity of the microenvironment.

## Author contributions

All authors listed have made a substantial, direct and intellectual contribution to the work, and approved it for publication.

### Conflict of interest statement

The authors declare that the research was conducted in the absence of any commercial or financial relationships that could be construed as a potential conflict of interest.
